# Two-Dimensional Nanosheet-Based Photonic Nanomedicine for Combined Gene and Photothermal Therapy

**DOI:** 10.3389/fphar.2019.01573

**Published:** 2020-01-21

**Authors:** Na Yoon Kim, Sara Blake, Diba De, Jiang Ouyang, Jinjun Shi, Na Kong

**Affiliations:** ^1^ Center for Nanomedicine and Department of Anesthesiology, Brigham and Women's Hospital, Harvard Medical School, Boston, MA, United States; ^2^ Department of Chemical Engineering, Northeastern University, Boston, MA, United States

**Keywords:** 2D nanosheet, gene therapy, photothermal therapy, graphene oxide, black phosphorus, translational metal dichalcogenide

## Abstract

Two-dimensional (2D) nanosheets are characterized by their ultra-thin structure which sets them apart from their bulk materials. Due to this unique 2D structure, they have a high surface-to-volume ratio that can be beneficial for the delivery of various drugs including therapeutic DNAs and RNAs. In addition, various 2D materials exhibit excellent photothermal conversion efficiency when exposed to the near infrared (NIR) light. Therefore, this 2D nanosheet-based photonic nanomedicine has been gaining tremendous attention as both gene delivering vehicles and photothermal agents, which create synergistic effects in the treatment of different diseases. In this review, we briefly provide an overview of the following two parts regarding this type of photonic nanomedicine: (1) mechanism and advantages of nanosheets in gene delivery and photothermal therapy, respectively. (2) mechanism of synergistic effects in nanosheet-mediated combined gene and photothermal therapies and their examples in a few representative nanosheets (e.g., graphene oxide, black phosphorus, and translational metal dichalcogenide). We also expect to provide some deep insights into the possible opportunities associated with the emerging 2D nanosheets for synergistic nanomedicine research.

## Introduction

**Scheme 1:**
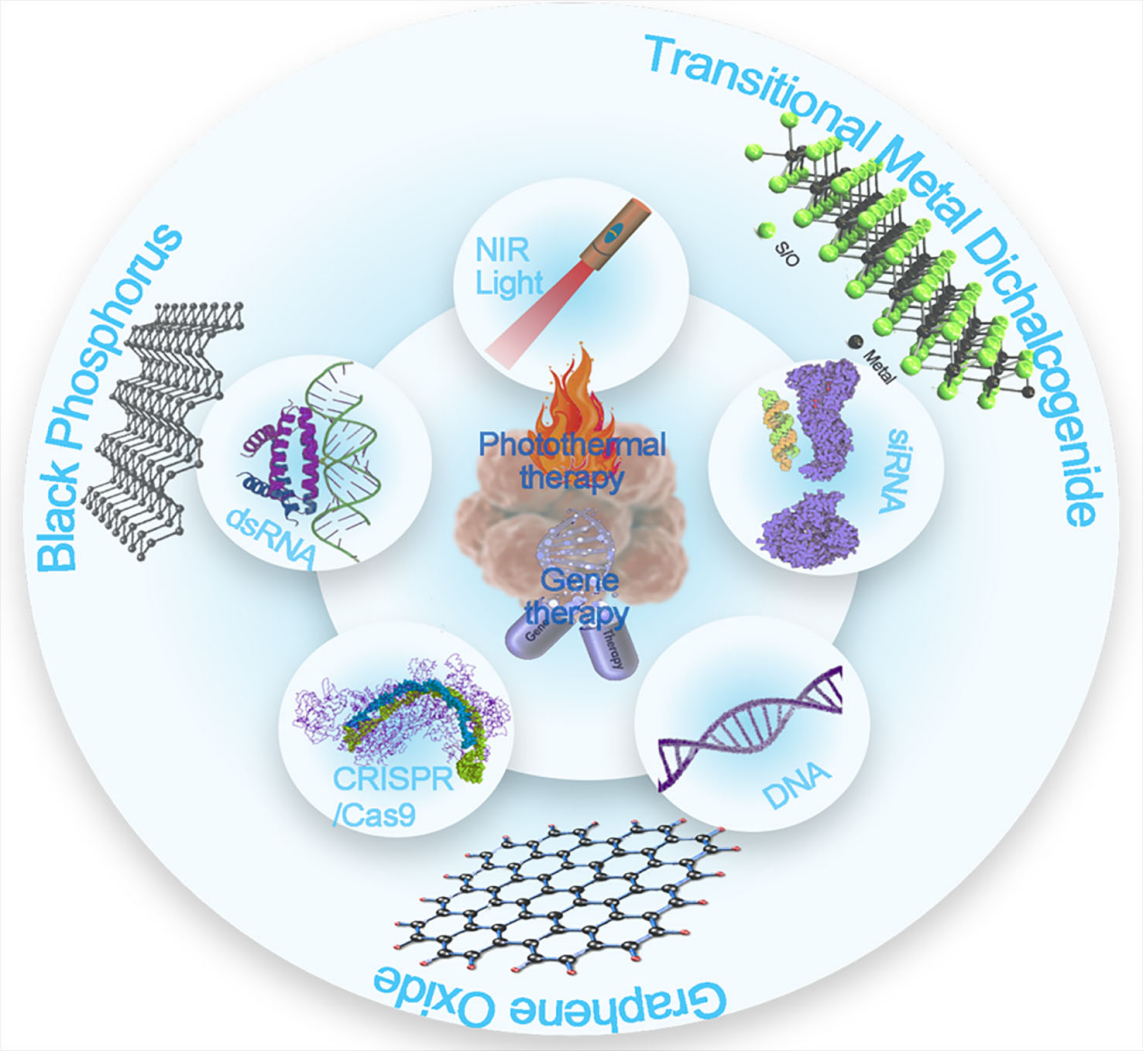
Overview of the types of 2D nanosheets that perform combined gene and photothermal therapy.

Two-dimensional (2D) nanosheets have gained great attention in the science community since the finding of graphene in 2004 (Novoselov, 2004). Since then, various 2D nanosheets consisted of different elements other than carbon have been additionally discovered, such as transition metal dichalcogenide, metal-organic framework, emerging monoelemental nanosheets (e.g., black phosphorus) and MXenes (Furukawa et al., 2013; Brent et al., 2014; Manzeli et al., 2017; Tao et al., 2019). 2D nanosheets have unique properties that can be useful in various fields of biomedical research, including nucleic acid delivery for gene therapy. Some 2D nanosheets are made of elements widely present in the human body, such as phosphorus, which makes them interesting as a delivery vehicle with potential biocompatibility and safety (Choi et al., 2018). So far, extensive studies on the use of 2D nanosheets in drug delivery have been done, and the variety of their applications have been demonstrated; these include gene delivery, small molecule drug delivery (Tao et al., 2017b; Ji et al., 2018 Tao et al., 2018), photothermal therapy (PTT) (Tao et al., 2017a; Xie et al., 2018; Ouyang et al., 2019), photodynamic therapy (PDT) (Ji et al., 2019), bioimaging (e.g., magnetic resonance imaging (MRI), X-ray Computed Tomography (CT), and photoacoustic imaging), and so on (Kurapati et al., 2016). This review will focus on 2D nanosheet-mediated combined gene and photothermal therapy. Although there has been a review article about combined photothermal and gene therapy mediated by nanomaterials in general (Kim et al., 2016c), this is the first review article on specific 2D nanosheet-mediated combine gene and photothermal therapy to our knowledge. Both gene therapy and PTT have gained great attention as new approaches to treat different diseases such as cancers and cardiovascular diseases (Kosuge et al., 2012; Shi et al., 2017; Rosenblum et al., 2018; Shi et al., 2019). 2D nanosheets' superior ability to perform both gene and photothermal therapy simultaneously can synergistically increase their therapeutic effects through different mechanisms. **Scheme 1** summarizes the content of this article. The structure of our article composes by the following two parts: (i) discussion on the mechanism and advantages of using nanosheets in gene therapy and photothermal therapy; and (ii) discussion on the mechanism and cases of nanosheet-mediated combined gene and photothermal therapy. We expect that this review to provide some perspectives for the furture applications of emerging 2D nanosheets in synergistic nanomedicine research.

### Gene Therapy

#### Mechanism

Successful gene delivery to a therapeutic target is a crucial step in gene therapy. Generally, gene delivery using nanomaterials follows the same path: cellular uptake and endosomal escape. One kind of endocytosis, pinocytosis has been considered a major way for nanomaterials to be taken up by cells (Sahay et al., 2010). Pinocytosis is a mechanism that cells use to uptake small molecules in the extracellular fluid and can be divided into four types: micropinocytosis, clathrin-mediated, caveolin-mediated, and clathrin-and-caveolae independent endocytosis (Conner and Schmid, 2003). Study done by Huang et al. provides a unique insight of what type of pinocytosis occurs during cellular graphene oxide-based sheet uptake. Huang et al. (2012) studied the cellular uptake of gold nanoparticle-loaded graphene oxide, 100–200 nm, in the presence of inhibitors of different endocytic mechanisms, such as amiloride (actin inhibitor), chlorpromazine (clathrin inhibitor), methyl-beta-cyclodextrin (caveolae inhibitor), and sodium azide (interfere endocytosis by depleting ATP). The surface-enhanced Raman scattering (SERS) analysis on Ca Ski cells showed that graphene oxide nanosheet uptake was substantially decreased when cells were treated with chlorpromazine, and sodium azide, while only a slight decrease in cellular uptake was observed in the groups treated with amiloride and methyl-beta-cyclodextrin. The results indicated that cellular uptake of graphene oxide nanosheets was clathrin and energy (ATP) dependent. According to Linares et al, macropinocytosis was a general mechanism for internalizing graphene oxide modified with fluorescein isothiocyanate and polyethylene glycolamine (PEG) FITC-PEG-GOs for Saos-2 osteoblasts, HepG2 hepatocytes, and RAW 264.7 (Linares et al., 2014). Interestingly, Mu et al. reported that large nanosheets can be uptaken by cells through phagocytosis as well (Mu et al., 2012). They synthesized protein-coated GO nanosheets with a diameter of 0.86 μm and an average height of 5.2 nm, and the formed GO nanosheets were mainly taken up by phagocytosis than clathrin-mediated endocytosis. The studies above demonstrated various possible pathways cells can uptake nanosheets, which is the first step in gene delivery.

Another important aspect of nanosheet-mediated gene delivery is its ability to perform endosomal escape. When nanosheets enter cells through endocytosis, the endocytic vesicles are formed, and the nanosheets will be sequentially passed to early endosome, late endosome/multivesicular body, and eventually to the lysosome, where proteins and nucleic acid are hydrolyzed by enzymes (Luzio et al., 2014). Based on the understanding of this process, two requirements in gene delivery cargo can be reasonably concluded: a gene carrier should be able to protect loaded genetic materials from degradation and be capable of escaping the endosome before they get to lysosome so the genetic materials can be safely delivered to the site of action, such as the cytoplasm for siRNA/mRNA or the nucleus for DNA. Numerous studies have proven that nanosheets can perform both roles successfully in different cell lines (Bao et al., 2011; Kou et al., 2014). Cationic polymers, such as polyethyleneimine (PEI) that are commonly used to load genes on 2D nanosheets causes ‘proton sponge effect', which induces the endosomal escape (Boussif et al., 1995). As Behr and Varkouhi reported, the cationic polymers induce proton accumulation in the endosome, which causes osmotic swelling and endosome rupture (Varkouhi et al., 2011). Through this mechanism, the gene carrying 2D nanosheets can successfully escape the endosome and perform transfection at the site of action, either in the cytoplasm or nucleus.

#### Loading Efficiency

One of the distinctive characteristics of nanosheets is its high surface to volume ratio due to its extremely thin structure. This allows nanosheets to load more drugs or genes than any other drug delivery methods. According to Peng et al. (2018), doxorubicin and indocyanine green loaded Gd3+ doped monolayered-double-hydroxide nanosheets showed an extremely high drug loading of 797.36% and the encapsulation efficiency of 99.67%, which is the highest drug loading content that was among reported drug delivery systems at the time.

Nanosheets' unique geometry allows efficient loading of genetic materials. Ji et al. reported an interesting statistic that compared the DNA loading efficiency of nanoparticles and nanosheets that were made of the same material, silica (Ji et al., 2017). The loading efficiency of silica nanoparticles was 3.3% while the loading efficiency of silica nanosheets was 34%. More studies on silica spheres are also worth noting (Li et al., 2017). The fact that the loading efficiency of silica nanosheets was more than ten times that of nanoparticles implies that the geometry of nanosheets plays an important role in increased loading capacity of genetic materials.

The gene loading capacity of nanosheets can also be significantly increased *via* chemical modifications. One of the most commonly used polymers to load genetic materials to nanosheets is polyethyleneimine (PEI). Polyethyleneimine is a branched polymer that has a positive charge, which makes it capable of electrostatically attracting negatively charged genetic materials like DNA and RNA. For example, Kou at el. reported MoS2 nanosheets modified with PEI and polyethylene glycol (PEG), Mos2-PEG-PEI, as a siRNA delivery vehicle (Kou et al., 2014) successful loading of siRNA to MoS2-PEG-PEI nanosheets was confirmed with an agarose gel electrophoresis assay. Teimouri et al. also reported graphene oxide nanosheets modified with different kinds of cationic polymers, such as polypropylenimine (PPI) and polyamidoamine (PAMAM) (Teimouri et al., 2016). Graphene oxides modified with either polymers, PPI or PAMAM, successfully performed transfection of murine neuroblastoma cells using plasmid GFP DNA. However, the transfection efficiency of PEI modified nanosheets were higher than those of PPI or PAMAM modified sheets.

#### Different Genetic Materials (DNA, dSRNA (siRNA), Oligonucleotides, Aptamers, CRISPR)

Since the discovery of nanosheets, it has been reported that numerous genetic materials can be loaded to nanosheets and delivered to the living cells for transfection. **Table 1** shows the representative examples of genetic materials that were reported to be successfully delivery by nanosheets. Mitter et al. reported dsRNA carrying layered double hydroxide (LDH) clay nanosheets that achieved sustained release of the genes on a plant, *Arabidopsis* seedlings, for more than 30 days (Mitter et al., 2017). Zhou et al. reported for the first time, the delivery and release of CRISPR/Cas9 complex through black phosphorus nanosheets (Zhou et al., 2018). The delivered Cas9N3-BPs showed successful genome editing and gene silencing both *in vitro* and *in vivo*. Various genetic materials could be delivered at once as well. Plasmid (pGL3), slice correction oligonucleotides (SC) or small interfering RNA (siRNA), was successfully loaded to cell penetrating peptide (PepFect 14) modified graphene oxide nanosheets, and great transfection efficacy was demonstrated on HeLa cells and U-87 MG-luc2 cells (Dowaidar et al., 2017). The delivery of siRNA could also open up the possibility of further applications of 2D nanosheets in immunotherapy *via* gene modulations (Xu et al., 2014; Li et al., 2016; Kwak et al., 2017). Wang et al. reported DNA/RNA aptamer loaded graphene oxide nanosheets that can sense ATP, GTP, adenosine derivatives, and guanosine derivatives (Wang et al., 2013). The loaded aptamers were labelled with fluorescent dye, and the successful delivery of the aptamers was shown *via* the analysis of fluorescence. Huang at el. reported a way to achieve transfection *via* naked DNA on silica glass nanosheets (Huang et al., 2015). This method had unique advantages in a sense that no vector was needed, and naked DNA was able to be transferred into cells that were known to be difficult to transfect, which like stem cells. Ji et al. introduced a new DNA delivery substrate using silica upright nanosheets (Ji et al., 2012). GFP reporter DNA complex was immobilized on the silica surface, which allowed a successful transfection on the embryonic kidney cell (HEK293XL). As demonstrated above, various kinds of genetic materials, such as DNA, RNA, aptamers, CRISPR/Cas9 complex, were successfully loaded to nanosheets and performed gene therapy on cells.

**Table 1:** Representative examples of genetic materials that were reported to be successfully delivery by nanosheets.

Material	Cell	Different Genetic Materials	Reference
Layered double hydroxide clay nanosheets	*Arabidopsis* seedlings	dsRNA	(Mitter et al., 2017)
Black phosphorus nanosheets	MCF-7	CRISPR/Cas9	(Zhou et al., 2018)
Graphene oxide nanosheets	HeLa	siRNA, plasmid, oligonucleotides	(Dowaidar et al., 2017)
Graphene oxide nanosheets	MCF-7	DNA/RNA aptamer	(Wang et al., 2013)
Silica nanosheets	Human embryonic kidney cells	DNA	(Ji et al., 2012)

### Photothermal Therapy

#### Mechanism

Photothermal therapy (PPT) is a recently developed therapy that uses heat produced by optical materials upon near-infrared (NIR, 700–1,000 nm) irradiation. Small sized nanosheets tend to accumulate at tumor sites when injected intravenously in animal models due to the enhanced permeability and retention effect (EPR effect) (Fang et al., 2011). Upon NIR irradiation, nanosheets absorb photons, the light energy is converted to heat energy, and the high temperature (about 42 degree Celsius) at tumour sites will cause tumor cell death (Shanmugam et al., 2014). As human tissues don't easily absorb NIR irradiation but nanosheets have excellent optical properties that effectively absorb the NIR irradiation, the PPT using nanosheets can effectively induce local temperature, which leads to selective killing of tumor cells (Zhang et al., 2019; Song et al., 2016; Song et al., 2019; Yin et al., 2019). Various studies have shown that previous studies have demonstrated that variety of nanosheets (graphene based, black phosphorous, transition-metal dichalcogenides, transition metal oxide, and MXenes) can effectively perform photothermal therapy selectively on cancer cells both *in vivo* and *in vitro* (Zhang et al., 2016; Ren et al., 2016; Yang et al., 2018).

#### Photothermal Conversion Efficiency and Stability

Photothermal conversion efficiency is a value that shows us how effectively a material converts light energy to thermal energy. This is an important factor in PPT because materials with high photothermal conversion efficiency can effectively produce heat to kill cancers with a certain NIR irradiation. One of the most commonly used equations to calculate photothermal conversion efficiency is the following (Liu et al., 2013):

$$\eta =\frac{\mathrm{hA}\Delta T_{\max}-Q_{s}}{I(1-10^{-A_{\lambda }})}$$

where *η* is the heat transfer coefficient, *A* is the container surface area, Δ*T_max_* is the maximum temperature change in the solution, *A*_λ_ is the 808nm light absorbance, and *I* is the laser power. However, so far, the limited photothermal conversion efficiency of PTT performing particles has been a challenge. For example, an organic photothermal agent, indocyanine green (ICG) dye, reported 3.37% photothermal conversion efficiency and 8.99% when it was in liposomal forms (Yoon et al., 2017). Based on recently reported studies, many of the synthesized nanosheets have exhibited high photothermal conversion efficiency. Fu et al. reported 2D MOS2 nanosheets that had a photothermal conversion efficiency of 62% (Fu et al., 2018). As Ren has reported in SnS nanosheets, the nanosheet structure (36.1%) enhances photothermal conversion efficiency when compared to that of the bulk (24%) (Ren et al., 2016). Wang et al. explains their MnO2 nanosheets' high photothermal conversion efficiency through the concept of oxygen vacancy where the photogenerated electron can be trapped and enhance the photothermal conversion (Wang et al., 2019).

## Synergistic Effects of 2D Nanosheets in Combined Gene in Photothermal Therapy

### Mechanism of Synergistic Effects of Combined Gene and Photothermal Therapy

The concept of synergistic effects between gene and photothermal therapy is based on the fact that nanosheets can perform two functions simultaneously. There are two main ways that the synergistic effects are achieved: (1) therapeutic effects from gene therapy and photothermal therapy occurring simultaneously or (2) increased therapeutic effects of gene therapy through thermal effects. Many cancer related studies demonstrated the first type of synergistic effects through the delivery of therapeutic genetic materials, meant to silence genes responsible for cancer proliferation promotion or chemo resistance, in combination with photothermal therapies that kill cancer cells via high temperature. The multi-functioning nanosheets that can perform both therapies showed higher tumor inhibitory affects when compared to the single therapy treated groups. The second type of synergistic effects is achieved through mild heat that promotes transfection through mechanisms like inducing endosomal escape or enhancing cell membrane permeability. This improves the rate and extent of transfection which leads to more effective delivery of genes. The cases of both types of synergistic effects are further discussed in detail below. To our knowledge, nanosheet-mediated combined gene and photothermal therapies were reported in three groups of nanosheets: graphene oxide, black phosphorus, and transitional metal dichalcogenide.

### Nanosheet-Mediated Combined Gene and Photothermal Therapy

#### Graphene Oxide

Graphene is the first discovered 2D nanomaterial that is made of carbon. Since the discovery, more members of the graphene family have been discovered, such as graphene oxide (GO), reduced graphene oxide(rGO), and few-layer graphene (Ge et al., 2018). Graphene oxide especially gained significant attention in drug delivery due to its hydrophilic nature and versatile surface chemistry, which can increase its biocompatibility (Reina et al., 2017). According to the distribution and biocompatibility study done by Zhang et al., graphene oxide showed good biocompatibility with red blood cells, which demonstrated their potential for intravenous drug delivery (Zhang, 2011).

**Figure 1:**
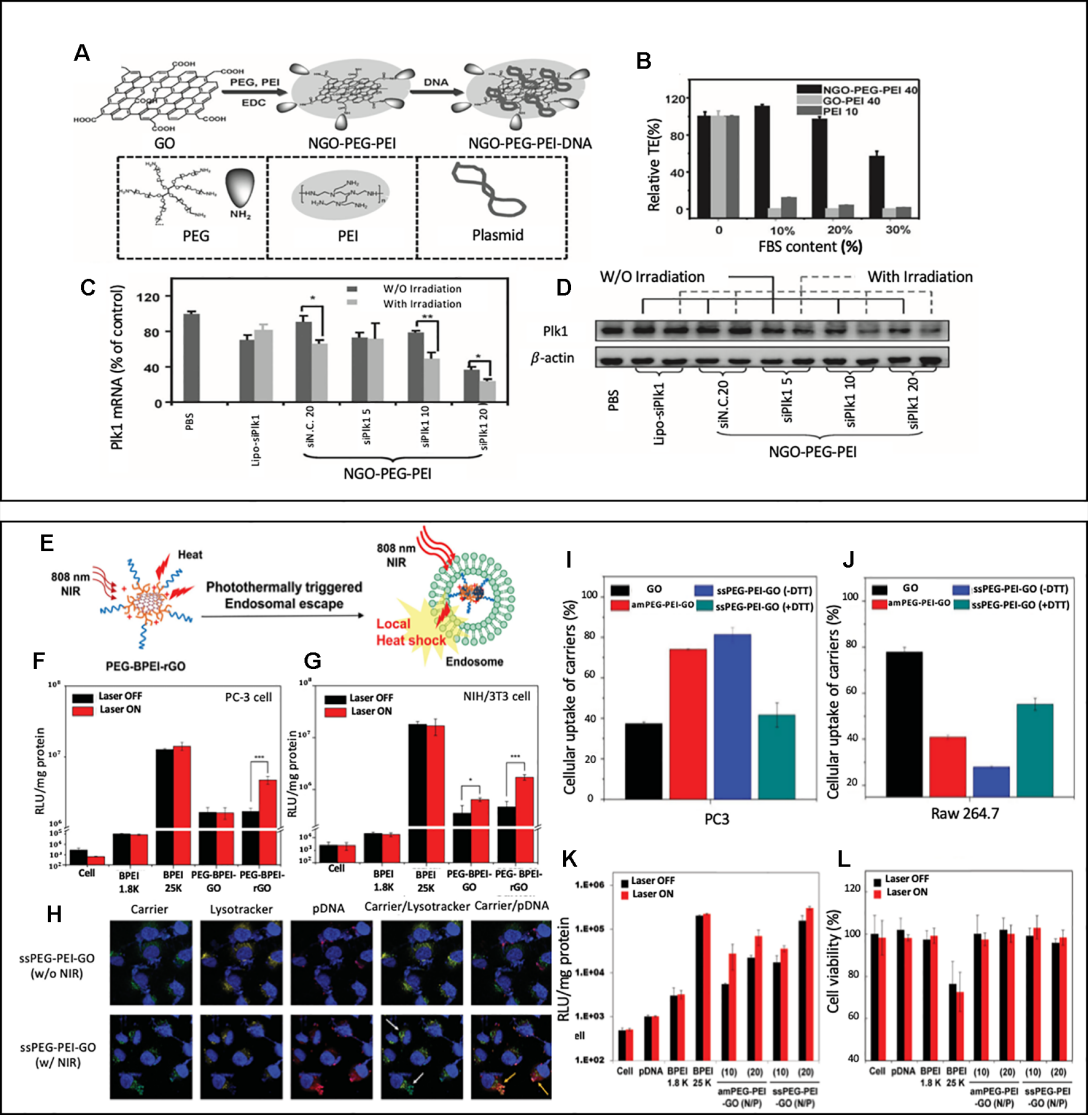
**(A)** A schematic illustration showing the synthesis of NGO-PEG-PEI/pDNA complex **(B)** Transfection efficiencies of NGO-PEP-PEI, GO-PEI and bare PEI transfected HeLa cells with various FBS contents **(C**, **D)** The expression levels of Plk1 mRNA **(C)** and protein **(D)** as determined by qRT-PCR and western blotting, respectively, in MDA-MB-435s cells after transfection. Reprinted with permission (Feng et al., 2013). Copyright 2013, John Wiley and Sons. **(E)** Schematic illustration of photothermal transfection mechanism. **(F**, **G)** Transfection of the BPEI (1.8 K and 25 K), PEG-BPEI-GO and PEG– BPEI–rGO with/without NIR irradiation in PC-3 and NIH/3T3 cell lines. Reprinted with permission (Kim and Kim, 2014). Copyright 2014, John Wiley and Sons. **(H)** Confocal fluorescence microscopic images of PC-3 cells treated with GO nanocarrier/pDNA complexes at various conditions. Nuclei (blue). Carrier and pDNA (green). TOTO (red). endo/lysosomes (yellow). Relative cellular uptake of GO, amPEG-PEI-GO, ssPEG-PEI-GO, and DTT-treated ssPEG-PEI-GO in **(I)** PC-3 and **(J)** Raw 264.7 cells. **(K)** transfection efficiency and **(L)** cytotoxicity of ssPEG-PEI-GO by luciferase assay and MTT assay. Gene transfection and cell viability of the GO nanocarriers were performed at N/P ratios of 2, 5, 10, and 20 with or without NIR irradiation in PC-3 cell lines. Reprinted with permission (Kim et al., 2016a) Copyright 2016, John Wiley and Sons.

To our knowledge, the first study to report GO based nanocarriers that performed both gene and photothermal therapy was by Feng et al. (2013). This study was meaningful in a sense that this was the first attempt to utilize GO nanosheet's optical and thermal properties to enhance the effectiveness of gene delivery. GO nanosheets were modified with PEG and PEI *via* amide bonds, which formed a nano GO-conjugate, (NGO-PEG-PEI) (**Figure 1A**). As shown in **Figure 1B**, the transfection capability of NGO-PEG-PEI in the serum condition was tested by measuring the transfection efficiency of EGFP pDNA loaded NGO-PEG-PEI nanocomposites in HeLa cells at various serum (FBS) concentrations. While the transfection efficiency of the groups transfected with GO-PEI or PEI decreased drastically as the serum concentration increased from 0 to 10%, 20%, and 30%, the NGO-PEG-PEI nanocomposites consistently showed good transfection efficiency in the presence of serum, which demonstrates NGO-PEG-PEI nanocomposite's superior transfection capability and protection of the loaded genetic material in the serum condition. NGO-PEG-PEI nanocomposite was capable of not only delivering pDNA but also delivering siRNA. As shown in **Figures 1C** and **D**, MDA-MB cells were treated with Plk1 siRNA loaded NGO-PEG-PEI nanocomposites, and the Plk1 mRNA and protein levels were analyzed through qRT-PCR and western blot. According to the Plk1 mRNA quantification results, the siRNA loaded NGO-PEG-PEI with different N/P ratios (5, 10, 20) consistently showed a lower expression of Plk mRNA expression than the lipofectatmine mediated transfected group. The suppression of Plk mRNA and protein expression was more obvious with the NIR irradiation. Especially in the NGO-PEG-PEI treated groups that had high N/P ratios (10 and 20), the suppression of Plk mRNA and protein expression were significantly enhanced with NIR irradiation. It was noted that low power density NIR light enhanced transfection efficiency through (808 nm laser at 50 W cm^−2^ for 20 min) increasing intracellular uptake of NGO-PEG-PEI. The local heating of the cell membrane led to enhanced membrane permeability (Tian et al., 2011).

**Figure 2:**
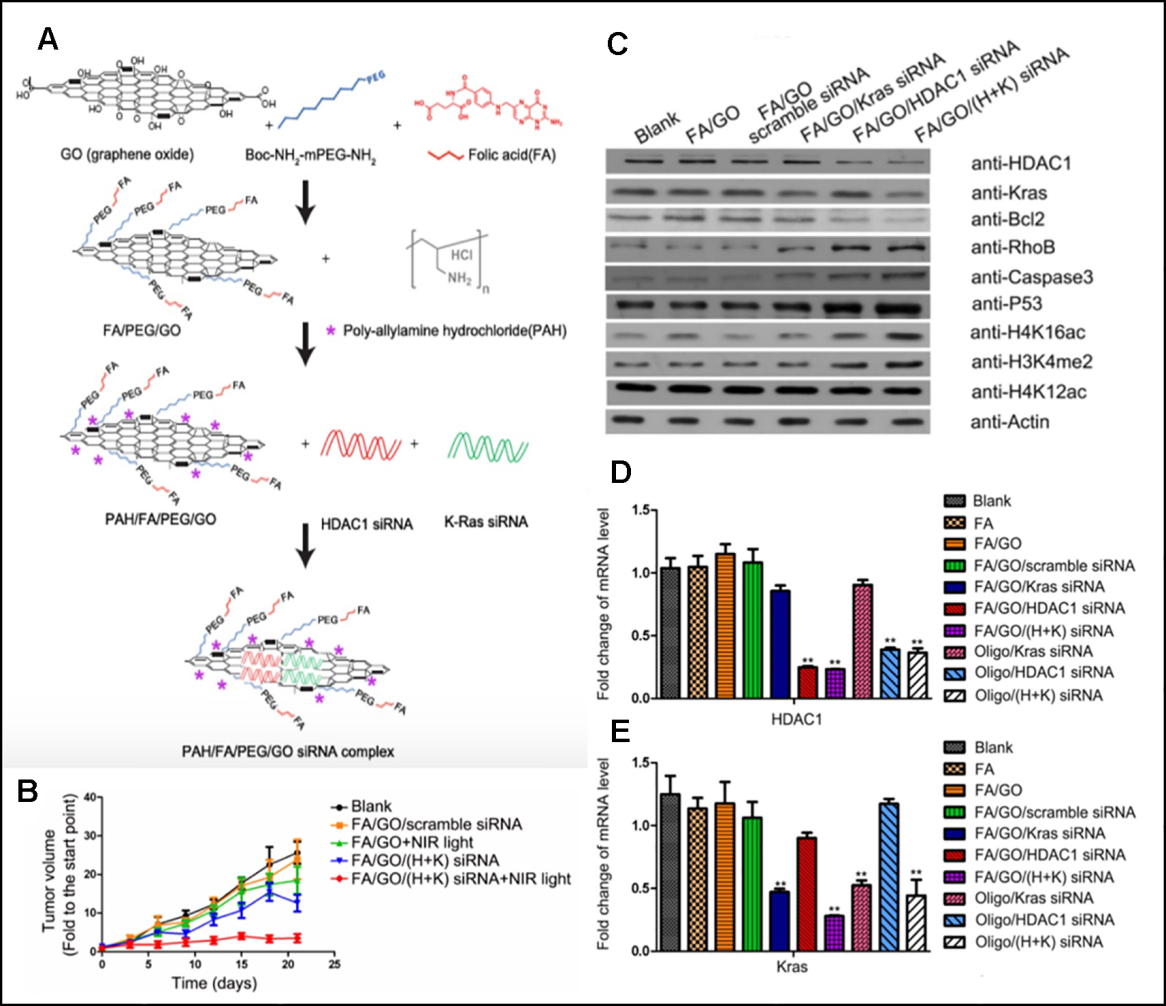
**(A)** Schematic overview of the FA/PEG/GO synthesis and gene loading process using engineered GO-based nanocarriers. **(B)** Relative changes in tumor volume over time. **(C**, **D**, and **E)** Target gene expression in MIA PaCa-2 cells treated with different nanoformulations *via* western blot for relative protein levels and RT-PCR for relative mRNA levels (Yin et al., 2017). Copyright 2017, Ivyspring International Publisher.

Another group that extensively studied GO nanosheet's potential to create synergistic effect from combining the photothermal effect and gene delivery was Kim's group. As illustrated in **Figure 1E**. Kim's group reported a reduced graphene oxide (rGO)-based gene delivery carrier modified with BPEI (low molecular-weight branched polyethyleneimine) and PEG, PEG-BPEI-rGO nanocomposites (Kim and Kim, 2014). For the evaluation of transfection efficacy of PEG-BPEI-rGO nanocomposites, luciferase gene expression assays were performed on PC-3 and NIH/3T3 cells using plasmid DNA (pDNA) carrying PEG-BPEL-rGO nanocomposites (**Figures 2F**, **G**) In both cell lines, the group exposed to the NIR irradiation (808 nm, 6 W/cm^2^, 20 min) showed 2–3-fold higher transfection efficiency than the group treated with PEG-BPEI-rGO without irradiation. The paper claimed that the NIR irradiation increased the temperature of intracellular PEG-BPEI-rGO, which induced the rupture of the endosomal membrane and lead to a higher release of DNA for transfection. The PEG-BPEI-rGO mediated gene delivery has a unique advantage as it achieves photothermally controlled gene delivery upon the NIR irradiation.

A different study performed by Kim's group introduced GO nanocarriers modified with BPEI and PEG *via* disulfide linkage, ssPEG-PEI-GO (Kim et al., 2016a). The main difference from the previous reported PEG-BPEL-rGO is that the disulfide bonds in ssPEG-PEI-GO nanocarriers enhanced their ability to be taken up by cancer cells and decreased their ability to be taken up by macrophages. **Figures 1I** and **J** showed cellular uptake of nanocarriers measured in PC3 (cancer cell) and Raw 264.7 (macrophage) with and without the presence of DTT, which reduces disulfide bonds. It is noted that the uptake of ssPEG-PEI-GO was higher than the unmodified GD in cancer cell but lower in macrophage, which showed its tumor targeting ability. **Figures 1K** and **L** evaluated ssPEG-PEI-GO nanocarrier's transfection efficiency. After PC3 cells were treated with pDNA carrying ssPEG-PEI-GO nanocarriers, the transfection efficiency with and without NIR irradiation was measured *via* luciferase assay. The transfection efficiency was increased up to five times with NIR irradiation compared to the group without irradiation, which confirms a photothermally enhanced transfection. The mechanism of photothermally enhanced transfection efficiency could be explained by two factors: NIR irradiation-induced endosomal disruption and reductive environment-induced gene release. **Figure 1H** showed an interesting confocal fluorescence images taken after the nanocarrier, pDNA, and endosome were labelled as FITC (green) TOTO (Red), and Lysotracker (yellow), respectively. Upon 10 minutes of NIR irradiation (808 nm laser at W cm^−2^), the endosomal escape of the carrier (green) and the following dissociation of pDNA (red) from the carrier was confirmed in confocal fluorescence images. This study had a significance in a sense that new chemical modifications using disulfide linkages were done on GO nanosheets to enhance the uptake by cancer cells while decreasing the uptake by macrophages.

Multifunctional GO nanosheets that can not only perform gene and photothermal therapy together but also have targeting ability for cancer was introduced by Yin et al. Their functionalized graphene oxide nanosheets (GO nanosheets) were able to perform tumor targeting, siRNA delivery, and photothermal therapy simultaneously (Yin et al., 2017). GO nanosheets were functionalized with folic acid (FA-mPEG-NH-Boc) for targeting of folate receptor-expressing cancer and coated with Poly-allylamine hydrochloride (PAH) (**Figure 2A**). PAH is a cationic polymer which can attract negatively charged siRNA *via* electrostatic interaction. Two types of siRNAs for oncogenes, HDAC1 siRNA and K-Ras siRNA, were loaded to GO nanosheets. After PaCa-2 pancreatic cancer cells were treated with synthesized GO sheet, the successful delivery of siRNA and gene silencing were confirmed by analysing the mRNA level of Kras and HDAC1 with RT-PCR and the protein levels with western blots (**Figures 2C**–**E**). It was noted that the transfection efficiency of nanosheets was higher than that of Oligofectamine, a commercial transfection agent. MIA PaCa-2 cells were planted subcutaneously to athymic nude mice for *in vivo* studies. Mice were treated with different treatments on a regular basis and were sacrificed on Day21 of the experiment. According to **Figure 2B**, mice with no treatment showed about 25 fold of initial tumor volume, FA/GO+NIR light treated group showed 18 fold of initial tumor volume, FA/GO/(HDAC1+Kas) siRNA treated group showed 10 fold of initial tumor volume, and mice treated with FA/GO/(HDAC1+Kas) siRNA+ NIR light showed almost no increase in tumor volume. These results clearly demonstrated the synergistic effect caused by the additive therapeutic effects from both gene therapy and photothermal therapy in suppressing tumor growth.

#### Black Phosphorous

Black phosphorous (BP) is a relatively new type of 2D nanosheet that has been extensively studied within the past few years since its discovery in 2014 (Li et al., 2014). It has been considered to have great potential for biomedical applications due to its biocompatibility. Phosphorus is a bone constituent that is present in our body and BP nanosheets can be degraded to phosphate and phosphonate, both are non-toxic to human body (Qian et al., 2017).

**Figure 3:**
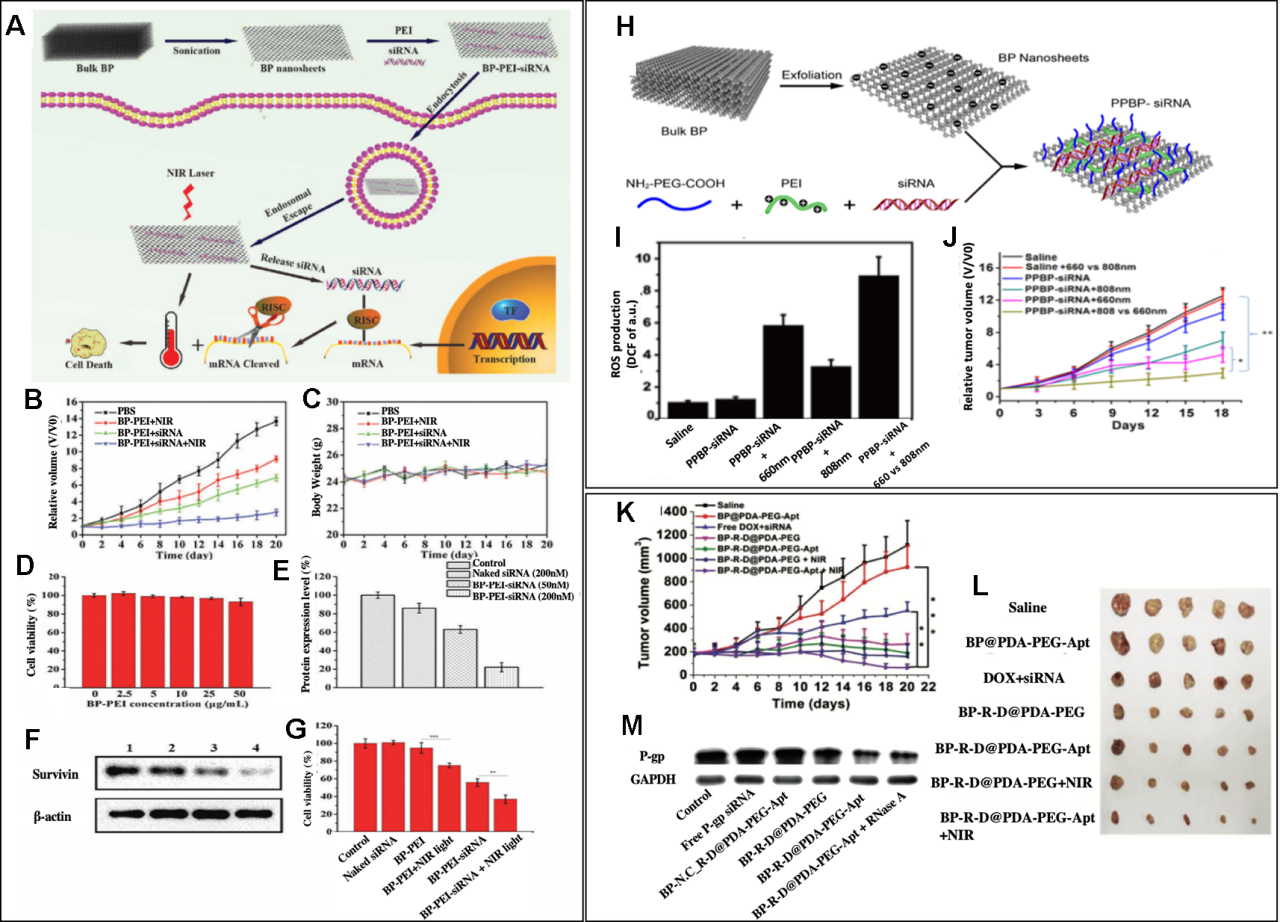
**(A)** Schematic illustration of the BP nanosheet-based siRNA delivery system for synergistic photothermal and gene therapy of cancer cells. **(B**, **C)** Growth curves of tumors and body weight of nude mice in different groups after various treatments (n = 5 for each group). **(D)** Cell viability assay after treating with different concentrations of BP-PEI for 24 h. **(E**, **F)** Western blot analysis of the survivin expression in MCF-7 cells. Lane 1: control; lane 2: naked siRNA (200 nM); lane 3: BP-PEI- siRNA (50 nM) and lane 4: BP-PEI-siRNA (200 nM). **(G)** Cell viability assay after treating with different formulations for 24 h. NIR light (808 nm NIR laser with a power density of 1.0 W cm^2^) was utilized to treat BP-PEI and BP-PEI-siRNA for 10 min. Reprinted with permission. (Wang et al., 2018) Copyright 2018, Royal Society of Chemistry. **(H)** Schematic illustration of the preparation of a BP nanosheet-based platform for siRNA delivery. **(I)** ROS production at the tumor site after different treatments (n = 3) **(J)** Tumor growth curves of subcutaneous HeLa xenograft in the different groups were measured. Compared with the curves in the 808 nm group and the two PBS groups, the tumor growth curve in the trimodal combined treatment group shows a significant difference (*p <0.05, **p <0.01, n = 5, respectively). Reprinted with permission (Chen et al., 2018). Copyright 2018, American Chemical Society. **(K)** Inhibition of tumor growth after different treatments. **(L)** Morphology of tumors removed from the sacrificed mice in all groups at the end point of study. **(M)** Western blot analysis. (Zeng et al., 2018). Copyright 2018, John Wiley and Sons.

To our knowledge, the first reported study of combined gene and photothermal therapy using BP nanosheets was by Wang et al. (2018). BP nanosheets were utilized as siRNA carriers by modifying BP nanosheets with positively charged polyethyleneimine (PEI), which allowed BP nanosheets to easily react with negatively charged siRNA molecules and form (BP-PEI-siRNA) (**Figure 3A**). Survivin protein expression in MCF-7 cells was significantly decreased after the cells were treated with BP-PEI-siRNA, which was confirmed by western blot analysis (**Figures 3F**, **E**). Both *in vitro* study and *in vivo* study were performed using MCF-7 cells to assess the synergistic effects of the combined siRNA therapy and NIR irradiation at 808 nm (PTT). In the *in vitro* cell viability study, shown in **Figure 3G**, the cell viability of MCF-7 cells was measured after 24 h treatment with BP-PEI+NIR light, BP-PEI-siRNA, and BP-PEI-siRNA + NIR light, which were about 70%, 50%, and 30%, respectively. **Figure 3B** showed the *in vivo* tumor growth study on MCF-7 bearing nude mice, the mice were divided into different treatment groups, and the relative volume of tumor (V/Vo) was measured on Day 20 of the treatment. On Day 20, the control group's relative tumor volume was 13 times of the initial volume while BP-PEI-siRNA+NIR treated group's relative tumor volume was only 2. NIR or siRNA treatment alone using BP-PEI+NIR or BP-PEI-siRNA showed antitumor effects to some extent (eight and six times of initial tumor volume on Day20), but significantly less effective than the combined gene and photothermal therapy. The body weights of all groups were comparable, which indicates the non-toxicity of the nanosheets (**Figure 3C**). **Figure 3D** also demonstrated the non-toxicity of the nanosheet treatment upto 50μg/mL *in vitro*. Wang's study held significance as it is the first reported study of combined gene and photothermal therapy using BP nanosheets to our knowledge and showed promising results of gene and photothermal therapy in both *in vitro* and *in vivo*. Due to the biodegradable nature of BP, clinical translation of nanocarriers consist of BP is promising.

Due to unique optical properties of BP nanosheets, not only photothermal therapy but also photodynamic therapy could be performed through using different wavelengths of light. Chen et al. reported BP nanosheets modified with NH2-PEG-COOH and PEI (PPBP) that can simultaneously perform gene therapy, PDT, and PTT (Chen et al., 2018). **Figure 3H** schematically showed the synthesis of siRNA loaded PPBPs. siRNA of human telomerase reverse transcriptase (hTERT), a gene that is closely related to tumor growth and metastasis, was loaded to PPBP nanosheets, which formed PPBP-siRNA (**Figure 3H**). HeLa cells were transfected with PPBP-siRNA for 48h, and an effective hTERT mRNA and protein suppression was shown *via* RT-PCR and western blot, respectively. PPBP-siRNA's great potential in PDT and PPT were also tested *in vitro*. The increased expression of a heat shock protein, Hsp70, upon exposure to 808 nm demonstrated PPBP-siRNA's ability to induce heat stress and perform PTT. PPBP-siRNA's ability to perform PDT was also confirmed by the intracellular ROS measurement after the irradiation at 660 nm (**Figure 3I**). An effective antitumor effect of gene, PDT, and PTT therapy was also confirmed *in vivo*, as shown in **Figure 3J**. HeLa tumor-bearing BALC/c nude mice were divided into different groups with different treatments, and the relative tumor volume (V/Vo) was measured until Day 18 of the treatment. On Day 18, the relative tumor volume of the control group, PPBP-siRNA treated group, PPBP-siRNA +808 nm group, PPBP-siRNA+ 660 nm group, and PPBP-siRNA + 808 nm + 660 nm group were 12, 10, 6, 4, 2 times of the initial tumor volume, respectively. PPBP-siRNA + 808 nm + 660 nm treated group showed the best antitumor effect in the *in vivo* study, which confirms an excellent synergistic effect of gene, PTT, and PDT therapy against cancer. This study highlighted BP nanosheets' advantageous characteristics in terms of its multifunctionality.

Fully utilizing the advantage of nanosheet's high loading capacity, Zeng et al. reported BP nanosheets that carry both chemodrug and genetic material and perform a photothermal therapy (Zeng et al., 2018). BP nanosheet-mediated chemo/gene/photothermal therapy showed a unique platform to overcome drug resistance in cancer. BP nanosheets were synthesized from bulk BP using a liquid exfoliation technique. Glycoprotein (P-gp) siRNA, which could downregulate permeability related glycoprotein on cancer cells and therefore help overcome drug resistance, was adsorbed onto the surface of BP nanosheets and doxorubicin (DOX) was loaded to BP nanosheets *via* electrostatic interactions. BP nanosheets were further modified by polydopamine (PDA) coating, and an aptamer conjugate, NH2-PEG-Apt was added to the PDA coating for tumor targeting purposes. The aptamer used was AS1411, which binds to nucleolin (NCL) overexpressed in cancer cells. The synthesized nanocomplex was called BP-R-D@PDA-PEG-Apt. The western blot analysis confirmed a successful downregulation of P-gp in BP-R-D@PDA-PEG-Apt treated MCF-7/MDR cells (**Figure 3M**). The antitumor efficacy of huhjBP-R-D@PDA-PEG-Apt was performed in a subcutaneous xenograft tumor model mice. As shown in **Figures 3K** and **L**, the DOX+siRNA group showed significantly less tumor growth inhibition compared to BP-R-D@PDA-PEG treated group, which indicated an important role of P-gp siRNA in overcoming drug resistance. Also, it was noted that NIR treated groups (BP-R-D@PDA-PEG-Apt +NIR and BP-R-D@PDA-PEG + NIR) consistently showed superior tumor growth inhibition effect than the groups treated with the same materials without NIR irradiation (BP-R-D@PDA-PEG-Apt and BP-R-D@PDA-PEG). While the saline treated group's tumor volume reached five times of the initial volume on Day 22 of the treatment, the tumor volume of the BP-R-D@PDA-PEG-Apt treated group was less than half of the initial tumor volume, which is less than ten time smaller than the control group. This showed great potential of black phosphorus nanosheets as a muti-platform to perform numerous therapies and overcome drug resistance simultaneously for maximized therapeutic effects.

#### Transitional Metal Dichalcogenide

Transitional metal dichalcogenides are semiconductors that can be named as MX2, where M is a transition metal and X is a chalcogen (Manzeli et al., 2017). The typical transition metal atom (M) includes Mo or W and chalcogen atom (X) includes Se and Se. Biomedical applications of transitional metal dichalcogenide have been hampered due to their low water solubility, non-uniformity, and colloidal stability, but extensive interest in TMD lead to the discoveries of new methods of synthesis, exfoliation, and surface modification that makes them more biocompatible (Agarwal and Chatterjee, 2018).

**Figure 4:**
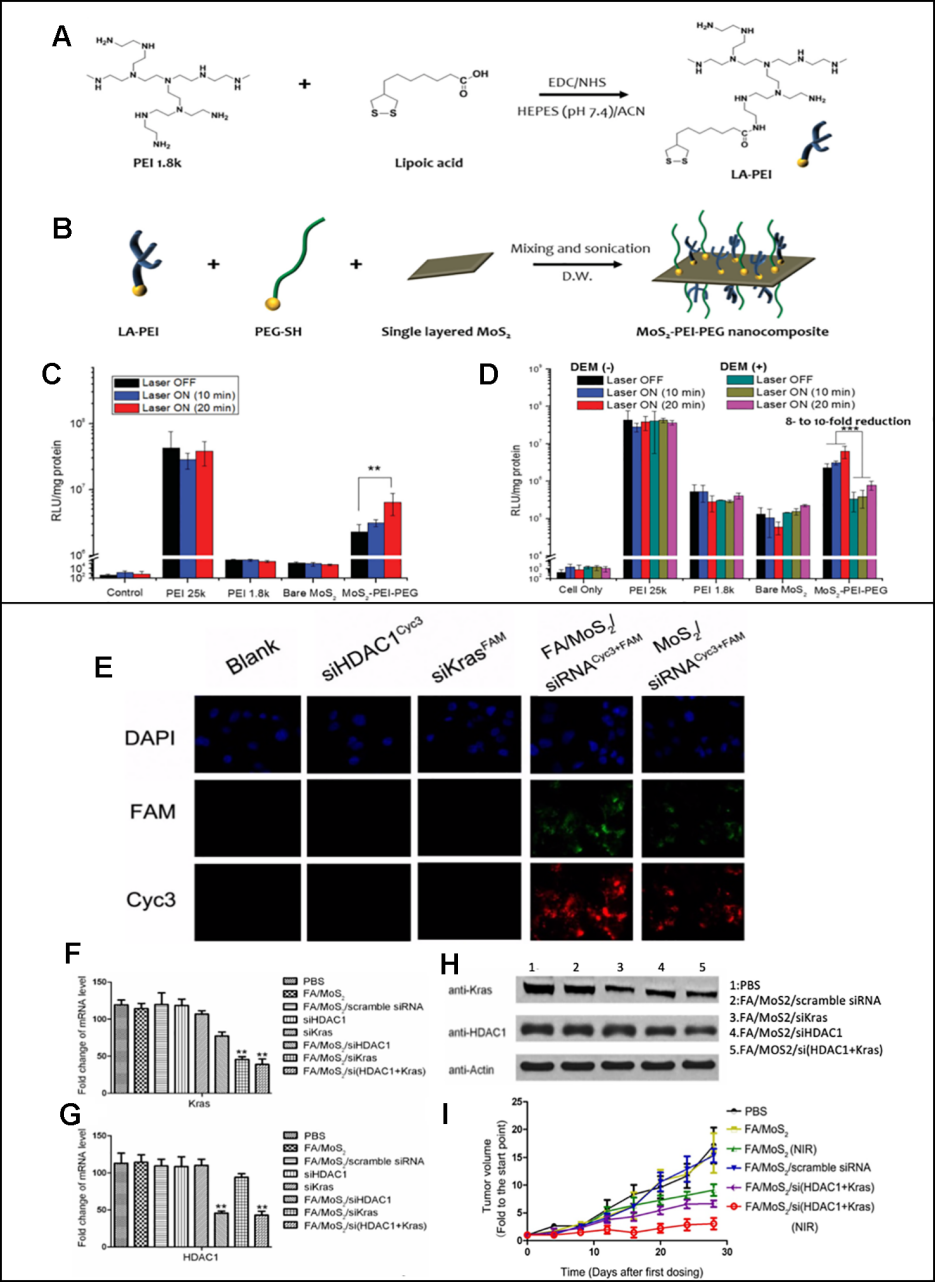
**(A)** Synthesis of LA–PEI through EDC/NHS amide coupling reaction. **(B)** Modification of MoS2 surface with LA–PEI and thiolated PEG (PEG–SH) by disulfide bond formation. Luciferase reporter gene expression assay of PEI 25k, PEI 1.8k, bare MoS2, and MoS2–PEI–PEG polyplexes in the absence and presence of NIR irradiation in **(C)** HCT 116 cells and **(D)** B16F1 cells at N/P ratio of 10. Reprinted with permission (Kim et al., 2016b). Copyright 2016, John Wiley and Sons **(E)** Fluorescent images of Panc-1 cells treated with different MoS2/siRNA nanocomplex formulations four hours after treatment. Nucleus (blue), FAM (green) and Cyc3 (red). **(F**, **G)** mRNA relative expression levels detected by RT-PCR. **(H)** Protein relative expression levels detected by Western Blotting. **(I)** Relative changes in tumor volume versus time of mice in different treatment groups (Yin et al., 2018). Copyright 2018, Ivyspring International Publisher.

One of the first reported MoS2 nanosheets that performed gene and photothermal therapy was reported by Kim et al. (2016b). They reported MoS2 nanosheets modified with PEI and PEG polymers *via* disulfide bonds. As shown in **Figures 4A** and **B**, lipoic acid conjugated to PEI, LA-PEI, was first synthesized and mixed with PEG-SH and single-layered MOS2 to synthesize MOS2-PEI-PEGs. The positive PEI polymer allowed electrostatic interatcion mediated loading of pDNA to the nanosheets, and the PEG polymers improved the biocompatibility and stability of nanosheets. Utilizing the MoS2 nanosheet's excellent photothermal conversion, photothermally triggered gene release in the cells was achieved upon the NIR irradiation. HCT116 cells were treated with luciferase gene loaded MoS2-PEI-PEG, and the luciferase reporter gene expression assay was carried out upon a mild NIR irradiation at 808 nm (2.5 W/cm^2^) for 0, 10 or 20 min. As the exposure time to NIR light increased, the increase in protein expression was observed in the MoS2-PEI-PEG cell (**Figure 4C**). An increased transfection upon the NIR irradiation was due to the local heat generation at the endosome, which causes the endosomal escape of the nanosheets. After the endosomal escape of pDNA-MoS2-PEI- PEG, polymer detachment and gene release were followed upon the exposure to the intracellular GSH that led to a reduction of disulfide bond of the nanosheets. The role of GSH in triggered gene release was confirmed by repeating the luciferase reporter gene expression assay in the presence of DEM, a chemical that inhibits GSH activity. According to **Figure 4D**, 8-10-fold reduction in protein expression was resulted, which confirmed the crucial role of GSH in triggered gene release. This study introduced a great potential of MoS2 nanosheets in performing photothermally controlled gene delivery.

While Kim et al. reported *in vitro* studies only, both *in vitro* and *in vivo* studies on the MoS2-mediated gene and photothermal combinatorial therapy was reported by Yin et al. (2018) MoS2 nanosheets modified with lipoic acid (LA), folic acid polyethyleneglycol polymer (FA-PEG), and polyallylamine hydrochloride (PAH). HDAC1 siRNAs and KRAS siRNAs were loaded to the modified MoS2 nanosheets. The overexpression of HDAC1 and mutant KRAS were known to cause proliferation of pancreatic cancer cells, and therefore silencing the two genes was used as an anti-cancer strategy in this study. As shown in **Figure 4E**, the fluorescence microscopy images were used to confirm a successful MoS2 mediated transfection of cyc3 labelled HDAC1 siRNA (red) and FAM labelled KRAS siRNA (green) in Panc-1 cells. The intensity of the fluorescence was stronger when MoS2 was modified with FA, which indicated effective targeted delivery of nanosheets. Decreased expressions of FAM and KRAS mRNA and protein were demonstrated *via* RT-PCR and western blot results (**Figures 4F**–**H**). Synergistic anti-cancer effects of gene and PTT was also shown *in vivo*. In animal studies, C57B1/6 mice were planted with Panc-1 cells and treated with different FA/MoS2 mediated therapies for 28 days. As shown in **Figure 4I**, for the groups treated with FA/MoS2 + NIR, FA/MoS2/si(HDAC1+Kras) and FA/MoS2/si(HDAC1+Kras) (NIR), the final tumor volumes were 8, 6, and 3 times of that of the original tumor volume while the control group's was 20 times of the original tumor. This study demonstrated that MoS2 nanosheet-mediated gene and photothermal therapy is a platform that was effective not only *in vitro* but also *in vivo*.

## Conclusion

2D nanosheets have unique and novel chemical properties that allow them to simultaneously perform photothermal and gene therapy. **Table 2** summarizes the examples of 2D nanosheet-mediated combined gene and photothermal therapy, mentioned in this review. Their high drug loading capacity for different therapeutics including nucleic acids and high photothermal conversion efficacy are advantageous in performing combined gene and photothermal therapies, which are shown to produce synergistic therapeutic effects through various examples. The limitations of nanosheet-mediated gene and photothermal therapy is that it is a relatively new concept, and there are not many reported cases that will help understand this field. The review paper was also limited to three groups of nanosheets that had the most reported cases of this synergistic therapy. Also, the mechanisms of synergistic effects were explained in two ways in the paper: additive therapeutic effects coming from both gene and photothermal therapy and increased therapeutic effect of gene therapy due to the temperature increase caused by the photothermal therapy. These two explanations are well studied and supported by other studies as well, but there could be more mechanisms behind the effect. The future researcher studying this field could focus on studying the exact and detailed pathways on the cause of the synergistic effects. The clinical transition of 2D nanosheets is yet to come as they are relatively new materials that require further studies in cytotoxicity, pharmacokinetics and biocompatibility. Future studies should address issues regarding validating the safety of nanosheets as drug delivery vehicles, identifying the specific need for both therapies, and assessing the side effects that could possibly arise from the interaction between the gene and photothermal treatment. The toxicity of 2D nanosheets largely varies based on numerous factors, including composition, size, shape, surface modification, and chemicals used in the synthesis process (Gurunathan and Kim, 2016). Through the optimization of these factors in designing nanosheets, biocompatible nanosheets are possible to be synthesized, which have been reported in numerous cases (Wang et al., 2015; Qu et al., 2017; Kaur et al., 2018). Considering the fact that multifunctional therapeutic modalities has been proposed as a way to tackle the problem of drug resistance in cancer, and both gene therapy and photothermal therapy have gained great attention in the past decade, 2D nanosheet-mediated combined gene and photothermal therapies may propose a possible alternative therapeutic modality in the future. Hopefully, this timely review would encourage future researchers to fully explore the potential of the 2D nanosheet-mediated combined gene and photothermal therapy.

**Table 2:** List of reported nanosheets that perform combined gene and photothermal therapy.

Material	Modified form	Functions of modification	Cell	Testing	Gene	Synergy mechanism	Reference
Graphene	GO-PEI-PEG	PEI (positive charge) PEG (stabilization)	HeLa	*In vitro*	Plk1 siRNA	PTT increase cell permeability which leads to effective transfection	(Feng et al., 2013)
	rGO-BPEI-PEG	BPE (positive charge) PEG (stabilization)	PC3 NIH/3T3	*In vitro*	Luciferase labelled pDNA	PTT facilitates endosomal escape	(Kim and Kim, 2014)
	GO-PEI-ssPEG	PEI (Positive charge) ssPEG (stabilization)	PC3 Raw 264.7	*In vitro*	Luciferase labelled pDNA	PTT facilitates endosomal escape	(Kim et al., 2016a)
	GO-PAH-PEG-FA	PAH (Positive charge) FA (targeting) PEG (stabilization)	MIA PaCa-2	*In vitro* *In vivo*	HDAC1 siRNA K-Ras siRNA	Synergistic effects in reducing tumor growth	(Yin et al., 2017)
Black Phosphorous (BP)	BP-PEI-PEG	PEI (positive charge) PEG (stabilization)	HeLa L549	*In vitro* *In vivo*	hTERT siRNA	PDT+PTT+genetherapy synergistic in reduced tumor growth ROS produced by PDT allows selective release of siRNA	(Chen et al., 2018)
	BP-PEI	PEI (positive charge)	MCF-7	*In vitro* *In vivo*	Survivin siRNA	Synergistic in reducing tumor growth	(Wang et al., 2018)
	BP-PDA-PEG	PDA (photothermal effects and stability) PEG (stabilization)	MCF-7 MCF-7/ADR	*In vitro* *In vivo*	P-gp siRNA AS1411 Aptamer (for targeting)	Drug+PTT+gene therapy: PTT induced drug release	(Zeng et al., 2018)
Transition Metal Oxide (TMO)	MoS2-PEI-PEG	PEI (positive charge) PEG (stabilization)	HCT116 B16F1	*In vitro*	Luciferase pDNA	PTT facilitates endosomal escape	(Kim et al., 2016b)
	MoS2-FA-PEG-PAH	PAH (positive charge) FA-PEG (binding to MoS2 and stabilization)	Panc-1	*In vitro* *In vivo*	HDAC1 siRNA K-Ras siRNA	Synergistic effects in reducing tumor growth	(Yin et al., 2018)

## Author Contributions

Conceptualization: NK and NYK. Writing—original draft: NYK and NK. Writing—review and editing: SB, DD, and JO. Supervision: NK and JS.

## Conflict of Interest

The authors declare that the research was conducted in the absence of any commercial or financial relationships that could be construed as a potential conflict of interest.

## FUNDING

This study is supported by the NEU CaNCURE program (NCI R25CA174650, NYK).
